# Impact of consensus guidelines for breast‐conserving surgery in patients with ductal carcinoma in situ

**DOI:** 10.1002/cnr2.1502

**Published:** 2021-07-10

**Authors:** Abigail Tremelling, Rebecca L. Aft, Amy E. Cyr, William E. Gillanders, Katherine Glover‐Collins, Virginia Herrmann, Julie A. Margenthaler

**Affiliations:** ^1^ Section of Surgical Oncology, Department of Surgery Washington University School of Medicine St Louis Missouri USA

**Keywords:** breast‐conserving surgery, ductal carcinoma in situ, margins

## Abstract

**Background:**

Consensus guidelines published in 2016 recommended a 2 mm free margin as the standard for negative margins in patients undergoing breast‐conserving surgery (BCS) for ductal carcinoma in situ (DCIS). The goal of the guideline recommendation was standardization of re‐excision practices.

**Aims:**

To evaluate the impact of this consensus guideline on our institutional practices.

**Methods:**

We identified all patients at our institution with pure DCIS who were initially treated with BCS from September 2014 to August 2018 using a prospectively‐maintained institutional database. A retrospective chart review was performed to determine margin status and re‐excision rates during the 2 years before and the 2 years after the guideline was published in order to determine the effect on our re‐excision rates. Close margins were defined as <2 mm.

**Results:**

In the 2 years before the consensus guideline was published, 184 patients with DCIS underwent BCS. Twenty‐six patients had positive margins and 24 underwent re‐excision, including three who had completion mastectomy. Of the remaining 159 patients, 76 had ≥2 mm (negative) margins. The remaining 82 patients had close margins and 48 of these patients (58.5%) underwent re‐excision, including one who had a completion mastectomy. Excluding the patients with positive margins, our re‐excision rate was 30.4% prior to the guideline. In the 2 years after the consensus guideline was published, 192 patients with DCIS underwent initial BCS. Twenty‐four patients had positive margins and 22 underwent re‐excision, including three who had completion mastectomy. Of the remaining 168 patients, 95 patients had ≥2 mm (negative) margins. The remaining 73 patients had close margins and 45 of those patients (61.6%) underwent re‐excision, including six who had completion mastectomy. Excluding the patients with positive margins, our re‐excision rate was 26.8% after the guideline.

**Conclusions:**

Our institution's re‐excision rate did not change significantly during the 2 years before and after the publication of the consensus guideline on adequate margins for patients undergoing BCT for DCIS. Our overall re‐excision rate decreased slightly. However, of the patients who had close margins, a larger proportion underwent re‐excision after the guideline was published. The guideline publication appears to have affected our institutional practices slightly, but not dramatically as many of our surgeons' practices were comparable to the guideline recommendations prior to 2016. We continue to use clinical judgment based on patient and tumor characteristics in deciding which patients will benefit from margin re‐excision.

## 1 INTRODUCTION

The approach to the treatment of breast cancer is an ever‐evolving field. The days of the Halsted radical mastectomy have long since been replaced with less invasive surgical options, including breast conservation surgery (BCS). A key component of BCS is obtaining adequate surgical margins so as to decrease the risk of ipsilateral breast tumor recurrence (IBTR).^1^, ^2^ Margin status for DCIS poses a unique challenge for the clinician due to the nature of the disease itself. DCIS often presents as a non‐palpable lesion identified as calcifications on screening mammography, and it can be multi‐focal with areas of normal ductal tissue between affected ductal segments.^3^, ^4^ For these reasons, the re‐excision rates have been reported as high as 70%, with an average re‐excision rate for DCIS around 30%–40%.^4^, ^5^, ^6^, ^7^

For many years, there was no clear definition of adequate margins for BCS. This resulted in variable practice patterns among providers across the country and within communities, and variable re‐excision rates.^8^, ^9^ In 2014, the Society of Surgical Oncology (SSO) and American Society of Radiation Oncology (ASTRO) published a consensus guideline on adequate margins for BCS with adjuvant whole breast irradiation (WBI) in patients with early stage invasive breast cancer. This statement recommended no ink on tumor as the standard for negative margins. These conclusions were based on a meta‐analysis of 33 studies including 28 162 patients, performed by a multi‐disciplinary expert panel, demonstrating no significant difference in IBTR for more widely clear margins.^10^

Similar guidelines were published in 2016 for DCIS, recommending 2 mm margins for BCS with planned adjuvant WBI. This guideline was released by the SSO, ASTRO, and the American Society of Clinical Oncology (ASCO). Once again, a multi‐disciplinary expert panel conducted a meta‐analysis of 20 studies including 7883 patients, demonstrating an optimal margin width of 2 mm to reduce the risk of IBTR. They also recommended the use of clinical judgment in determining the need for re‐excision in patients with negative margins less than 2 mm.^11^

Both of these society guidelines sought to standardize re‐excision practices for BCS in order to reduce re‐excision rates and their subsequent effects on patient outcomes while minimizing the risk of IBTR.^10^, ^11^ We previously reported on the potential impact of the 2014 guideline on our institution's practice, with an estimated reduction of over 5% in re‐excision rates for invasive disease.^12^ We now analyze the impact of the 2016 guideline at our institution by evaluating re‐excision rates before and after the publication of the consensus guideline for DCIS.

## 2 METHODS

### 2.1 Study population

Approval was obtained from the institutional review board at Washington University in St. Louis. Utilizing a prospectively maintained institutional database, all patients 18 years and older with a diagnosis of pure DCIS (no invasive component) undergoing BCS from September 2014 to August 2018 were identified. Only patients whose initial surgery was performed at Barnes Jewish Hospital/Siteman Cancer Center were analyzed. Patients were excluded if they had their index surgical procedure at an outside institution.

Patients were divided into two groups based on the date of their initial surgical procedure being before or after September 2016, when the SSO‐ASTRO‐ASCO guideline would have been adopted by our institution.

### 2.2 Data analysis

A retrospective chart review was performed to obtain demographic, clinical, and pathologic data from our electronic medical record. During the study period, six surgeons were practicing at our institution, at four different surgical centers, all sharing the same electronic medical record and institutional database. Surgical specimens were marked intraoperatively by the surgical team using a short stitch superiorly and a long stitch laterally. The breast pathology team then inked and processed the specimens according to institutional protocols. The final pathology reports were reviewed to determine margin status on all index and re‐excision procedures. Margins were defined as negative if they were ≥2 mm from the tumor, close if they were within 2 mm of the tumor, and positive if there was ink on the tumor. It is important to note that our institutional definitions of negative, close, and positive margins were ultimately the same as the guideline definition of these margins.

For each group, overall re‐excision rates were calculated as the percentage of patients undergoing any type of re‐excision procedure (including mastectomy) out of the total number of patients undergoing initial BCS. Re‐excision rates were similarly calculated for the subgroups of patients with close margins, with positive margins, and with non‐positive margins (i.e. excluding those with positive margins). Similar calculations were also performed after excluding those patients who did not have a pre‐operative diagnosis of DCIS but were found to have DCIS on final pathology. We defined the “true re‐excision rate” as the re‐excision rates excluding those patients who underwent excisional biopsies as the initial surgical procedure. Of note, positive posterior margins and positive anterior margins are routinely not re‐excised at our institution if the surgeon has documented resection to the pectoralis fascia or sub‐dermis, respectively. Statistical analyses were performed using a Fisher's exact test for re‐excision rates.

## 3 RESULTS

### 3.1 Study population

A total of 376 patients underwent initial BCS at our institution for pure DCIS during the four‐year study period. 184 of these patients had their initial surgery between September 2014 and August 2016, during the 2 years before the SSO‐ASTRO‐ASCO guideline was adopted by our institution. The remaining 192 patients had their initial surgery during the 2 years following the guideline adoption, from September 2016 to August 2018. Patient demographics and tumor characteristics were similar between the two groups (Table 1).

**TABLE 1:** Patient demographics and tumor characteristics

Variable	Pre‐guideline *n* = 184	Post‐guideline *n* = 192	*p* values
Mean age	60	59	*p* = .27
Race			*p* = .19
Caucasian	125	129	‐
Black/African American	53	52	‐
Asian	6	11	‐
Hormone receptor status			*p* = .45
ER+	166	169	‐
ER−	15	12	‐
Unknown	3	11	‐
Tumor grade			*p* = .11
1	41	32	‐
2	89	78	‐
3	54	82	‐

### 3.2 Pre‐guideline (September 2014 through August 2016)

**FIGURE 1:**
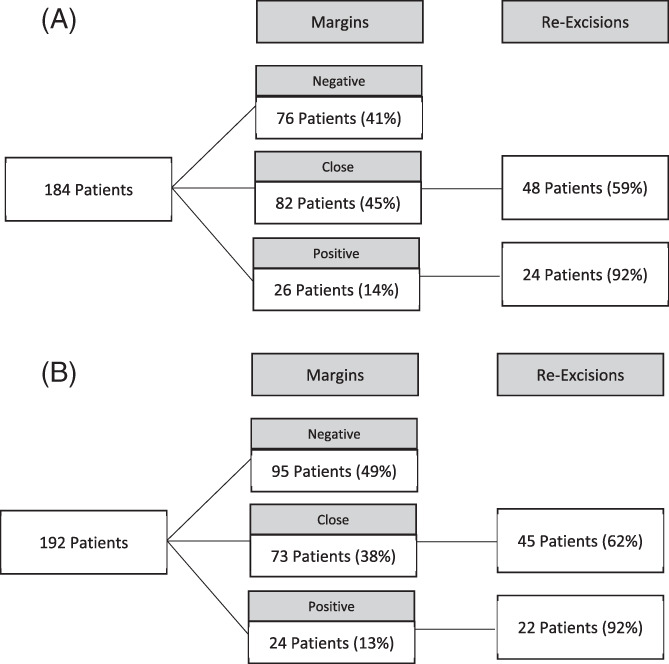
(A) Pre‐guideline index procedure margin status and re‐excisions. (B) Post‐guideline index procedure margin status and re‐excisions

Of the 184 patients in the pre‐guideline cohort, 76 patients (41.3%) had negative margins, 26 (14.1%) had positive margins, and 82 (44.6%) had close margins on initial excision. There were 8 patients who underwent an oncoplastic surgical technique with combined reduction mammoplasty and all had negative margins. Twenty‐four of the patients with positive margins (92.3%) and 48 of the patients with close margins (58.5%) underwent re‐excision, for an overall re‐excision rate of 39.1%. Patients with positive margins who did not undergo re‐excision had either positive posterior or anterior margins and the surgeon felt that they had resected to the pectoralis fascia or sub‐dermis, respectively. The reasons why patients with close margins were not re‐excised was difficult to ascertain from the retrospective review. Excluding the patients with positive margins, our re‐excision rate was 30.4%. This cohort included 43 patients who underwent excisional biopsy and were upgraded to a diagnosis of DCIS on final pathology. If we exclude these patients and calculate the re‐excision rates for only those patients with a pre‐operative diagnosis of DCIS, our true re‐excision rate was 32.6% (Figure 1A and Table 2).

**TABLE 2:** Re‐excision rates

	Pre‐guidelines *n* = 184	Post‐guidelines *n* = 192	*p* values
Overall	72/184 (39.1%)	67/192 (34.9%)	*p* = .48
Of close margins	48/82 (58.5%)	45/73 (61.6%)	*p* = .34
Of positive margins	24/26 (92.3%)	22/24 (91.7%)	*p* = .51
Excluding positive margins (Of non‐positive margins)	48/158 (30.4%)	45/168 (26.8%)	*p* = .27
True overall re‐excision rate (Excluding excisional biopsies)	46/141 (32.6%)	46/157 (29.3%)	*p* = .43

Twenty‐nine patients (40.3%) had residual DCIS identified on re‐excision. One patient had a second re‐excision procedure for persistently close or positive margins. Four patients ultimately had a mastectomy performed on subsequent excisions (three on the first re‐excision procedure and one on the second re‐excision). Details are displayed in Table 3.

**TABLE 3:** Re‐excision procedure outcomes

	Pre‐guidelines	Post‐guidelines
Residual DCIS on re‐excision	29/72 (40.3%)	27/67 (40.3%)
Second re‐excision procedure	1	7
Mastectomy on subsequent excision	4	9

At a mean follow‐up of 4.2 years, there have been 3 IBTRs in the pre‐guideline cohort. Two of these patients had an invasive recurrence, while the third patient had a new/recurrent DCIS. All three were patients who had initial positive margins and underwent re‐excision at the initial diagnosis. One additional patient in the pre‐guideline cohort experienced a new contralateral invasive breast cancer during follow‐up.

### 3.3 Post‐guideline (September 2016 through August 2018)

Of the 192 patients in the post‐guideline cohort, 95 patients (49.5%) had negative margins, 24 (12.5%) had positive margins, and 73 (38%) had close margins on initial excision. There were 13 patients who underwent an oncoplastic surgical technique with combined reduction mammoplasty and all had negative margins. Twenty‐two of the patients with positive margins (91.7%) and 45 of the patients with close margins (61.6%) underwent re‐excision, for an overall re‐excision rate of 34.9%. Patients with positive margins who did not undergo re‐excision had either positive posterior or anterior margins and the surgeon felt that they had resected to the pectoralis fascia or sub‐dermis, respectively. The reasons why patients with close margins were not re‐excised was difficult to ascertain from the retrospective review. Excluding the patients with positive margins, our re‐excision rate was 26.8%. This cohort included 35 patients who underwent excisional biopsy and were upgraded to a diagnosis of DCIS on final pathology. Excluding those patients, our true re‐excision rate was 29.3% (Figure 1B and Table 2).

Twenty‐seven patients (40.3%) had residual DCIS identified on re‐excision. Seven patients had a second re‐excision procedure for persistently close or positive margins. Nine patients ultimately had a mastectomy performed on subsequent excisions (six on the first re‐excision procedure and three on the second re‐excision). Details are displayed in Table 3.

At a mean follow‐up of 3.1 years, there have been no recurrences in the post‐guideline cohort.

All *p* values demonstrated no significant difference in the re‐excision rates in the pre‐ and post‐guideline cohorts (Table 2).

## 4 DISCUSSION

BCS has become the preferred surgical option in the surgical treatment of breast cancer for patients with early disease. However, the definition of clear margins for BCS has been one of contention since its introduction. With the development of consensus guidelines in 2014 and 2016 on margin recommendations for BCS, there are now well‐defined recommendations to aid in clinical decision making regarding re‐excision.

Although there were no significant differences in the rates of re‐excision before and after the 2016 guideline was adopted, our overall re‐excision rate trended down. Our re‐excision rates trended up for margins that were negative but less than 2 mm (close) before and after the guideline adoption (58.5% and 61.6%, respectively). Based on these findings, we conclude that our practice patterns for re‐excising margins in BCS for pure DCIS from 2014 to 2016 were already similar to the guideline recommendations. This is further supported by the fact that we did not see any re‐excisions for margins greater than 2 mm in the pre‐guideline group, again demonstrating that we were already using a margin threshold of 2 mm in most circumstances.

At our institution, individual cases are presented at a weekly multi‐disciplinary tumor board where we review the patient's images and pathology slides and discuss management recommendations. This venue is often used to discuss adequacy of margins and the need for additional surgery based on the patient's age, life expectancy, co‐morbidities, extent of disease, cosmetic outcome, and risk of recurrence. We have in the past used, and continue to use, clinical judgment to determine if an individual patient will benefit from margin re‐excision.

In both groups, all patients with positive margins that did not undergo re‐excision had documentation for the reason no re‐excision was performed (i.e. margin at the level of the skin or pectoralis fascia, focally positive margin, and no residual calcifications on mammogram). For patients with close margins that were not re‐excised, we found similar documentation in 4 of the 35 patients (11.4%) in the pre‐guideline cohort, and in 8 of the 28 patients (28.6%) in the post‐guideline cohort. This demonstrates that although we may have followed similar practices before the guideline publication, our documentation in support of our clinical decision‐making began to improve.

The strengths of our study are in our patient population and inclusion criteria. Both groups had similar patient and tumor characteristics. We included all patients with a diagnosis of pure DCIS undergoing initial BCS at our institution. By excluding patients with DCIS found on excisional biopsy in our subgroup analysis, we were able to determine our true re‐excision rates for those patients whose initial surgery had the intent of obtaining clear margins.

Our study is limited as it is a retrospective review from a single institution. Additionally, we did not account for variability in practice patterns between individual surgeons at our institution, such as operative technique and personal differences in re‐excision thresholds. For example, the use of selective versus routine cavity shave margins during the study period varied among surgeons. These practices may have affected re‐excision rates between surgeons, but likely would have been the same in the pre‐ and post‐guideline cohorts.

In conclusion, our findings demonstrate that our institution's practice patterns were similar during the 2 years before and after the publication of the 2016 SSO‐ASTRO‐ASCO consensus guideline on margins in BCS for patients with DCIS. Our re‐excision rates did decline slightly, and we continue to use clinical judgment based on patient and tumor characteristics in deciding which patients will benefit from margin re‐excision. Despite the current consensus guidelines, margin status will continue to be an area of debate and research in BCS as we try to find the optimal balance between less surgery and minimizing the risk of local recurrence.

## CONFLICT OF INTEREST

The authors have stated explicitly that there are no conflicts of interest in connection with this article.

## ETHICS STATEMENT

Ethical approval for this retrospective analysis was obtained by the Institutional Review of Board of Washington University School of Medicine. A waiver of consent was obtained.

## AUTHOR CONTRIBUTIONS

All authors had full access to the data in the study and take responsibility for the integrity of the data and the accuracy of the data analysis. *Conceptualization*, A.T. and J.A.M.; *Methodology*, A.T. and J.A.M.; *Investigation*, A.T. and J.A.M.; *Formal Analysis*, A.T. and J.A.M.; *Resources*, *all authors; Writing‐Original Draft*, A.T. and J.A.M.; *Writing ‐ Review and Editing, all authors; Visualization*, A.T.; *Supervision*, J.A.M.

## Data Availability

The data is available upon request.

## References

[1] Silverstein MJ , Lagios MD , Groshen S . The influence of margin width on local control of ductal carcinoma in situ of the breast. N Engl J Med. 1999;13:1455‐1461. 10.1056/NEJM199905133401902 10320383

[2] Dunne C , Burke JP , Morrow M , Kell MR . Effect of margin status on local recurrence after breast conservation and radiation therapy for ductal carcinoma in situ. J Clin Oncol. 2009;27(10):1615‐1620. 19255332 10.1200/JCO.2008.17.5182

[3] Marinovich ML , Azizi L , Macaskill P , et al. The association of surgical margins and local recurrence in women with ductal carcinoma in situ treated with breast‐conserving therapy: a meta‐analysis. Ann Surg Oncol. 2016;23:3811‐3821. 27527715 10.1245/s10434-016-5446-2 PMC5160992

[4] Toss MS , Pinder SE , Green AR , et al. Breast conservation in ductal carcinoma *in situ* (DCIS): what defines optimal margins? Histopathology. 2017;70:681‐692. 28000325 10.1111/his.13116

[5] Thomas J , Evans A , Macartney J , et al. Radiological and pathological size estimations of pure ductal carcinoma in situ of the breast, specimen handling and the influence on the success of breast conservation surgery: a review of 2564 cases from the Sloane Project. Br J Cancer. 2010;102(2):285‐293. 20051953 10.1038/sj.bjc.6605513 PMC2816666

[6] Langhans L , Jensen M‐B , Talman M‐LM , Vejborg I , Kroman N , Tvedskov TF . Reoperation rates in ductal carcinoma in situ vs invasive breast cancer after wire‐guided breast‐conserving surgery. JAMA Surg. 2017;152(4):378‐384. 28002557 10.1001/jamasurg.2016.4751 PMC5470426

[7] Jeevan R , Cromwell DA , Trivella M , et al. Reoperation rates after breast conserving surgery for breast cancer among women in England: retrospective study of hospital episode statistics. BMJ. 2012;345:e4505. 22791786 10.1136/bmj.e4505 PMC3395735

[8] Hughes L , Hamm J , Mcgahan C , Baliski C . Surgeon volume, patient age, and tumor‐related factors influence the need for re‐excision after breast‐conserving surgery. Ann Surg Oncol. 2016;23(Suppl 5):656‐664. 27718033 10.1245/s10434-016-5602-8

[9] Azu M , Abrahamse P , Katz SJ , Jagsi R , Morrow M . What is an adequate margin for breast‐conserving surgery? Surgeon attitudes and correlates. Ann Surg Oncol. 2010;17:558‐563. 19847566 10.1245/s10434-009-0765-1 PMC3162375

[10] Moran MS , Schnitt SJ , Giuliano AE , et al. Society of Surgical Oncology‐American Society for Radiation Oncology consensus guideline on margins for breast‐conserving surgery with whole‐breast irradiation in stages I and II invasive breast cancer. Ann Surg Oncol. 2014;21(3):704‐716. 24515565 10.1245/s10434-014-3481-4

[11] Morrow M , Van Zee KJ , Solin LJ , et al. Society of Surgical Oncology–American Society for Radiation Oncology–American Society of Clinical Oncology consensus guideline on margins for breast‐conserving surgery with whole‐breast irradiation in ductal carcinoma in situ. Ann Surg Oncol. 2016;23(12):3801‐3810. 27527714 10.1245/s10434-016-5449-z PMC5047939

[12] Yu J , Elmore LC , Cyr AE , Aft RL , Gillanders WE , Margenthaler JA . Cost analysis of a surgical consensus guideline in breast‐conserving surgery. J Am Coll Surg. 2017;225(2):294‐301. 28414115 10.1016/j.jamcollsurg.2017.03.020 PMC5663461

